# Prevalence and treatment patterns of adult atopic dermatitis in the UK Clinical Practice Research Datalink

**DOI:** 10.1002/ski2.232

**Published:** 2023-05-10

**Authors:** C. Elise Kleyn, Robert McKenzie, Alexandra Meeks, Beatrice Gittens, Lill‐Brith von Arx

**Affiliations:** ^1^ The Dermatology Centre The University of Manchester Manchester UK; ^2^ Eli Lilly and Company Ltd Basingstoke UK; ^3^ Eli Lilly and Company Indianapolis Indiana USA; ^4^ Eli Lilly and Company Copenhagen Denmark

## Abstract

The prevalence of active atopic dermatitis (AD) in adults in the UK according to disease severity shows variability. This study evaluated disease prevalence and treatment patterns among the adult UK population with AD. Data were obtained from the Clinical Practice Research Datalink (CPRD) database. Adults with active AD were identified by an AD‐related prescription or general practitioner visit within the same calendar year. Prevalence was defined as the number of patients with active AD on 1 January of each year as a percentage of the number of adults in the CPRD population on that date. Moderate‐to‐severe disease was classified as either referral to a specialist or prescription(s) for topical calcineurin inhibitors, phototherapy, or systemic treatment. Patient characteristics and treatment and referral patterns were analysed for patients with active AD in 2019. The overall prevalence of AD was stable at 2.4% per year during the period 2015–2019. In 2019, mean patient age (± standard deviation) was 52.6 ± 21.0 years, 58.2% of patients were female and mean disease duration was 9.4 ± 5.9 years. The most prescribed treatment was topical corticosteroids, in 78.5% of patients. 36.7% of patients with moderate‐to‐severe AD were prescribed systemic agents and 59.8% (vs. 32.3% of patients with mild AD) were referred to any secondary care or specialist treatment. The prevalence of active AD in the adult UK population was stable over the 5‐year period (2015–2019) and was comparable to estimates from similar studies based on UK primary healthcare records.

## Graphical abstract

Using data from the Clinical Practice Research Datalink database, the prevalence of active atopic dermatitis (AD) in the UK adult population was estimated to be stable at 2.4% per year during the period 2015–2019 and was comparable to estimates from similar studies based on UK primary healthcare records. Overall, 7.5%–8.3% of adults with AD had moderate‐to‐severe disease during the 5‐year period. In 2019, mean patient age was 52.6 years, 58.2% of patients were female, mean disease duration was 9.4 years and topical corticosteroids were the most prescribed treatment (in 78.5% of patients); 36.7% of patients with moderate‐to‐severe AD were prescribed systemic agents.

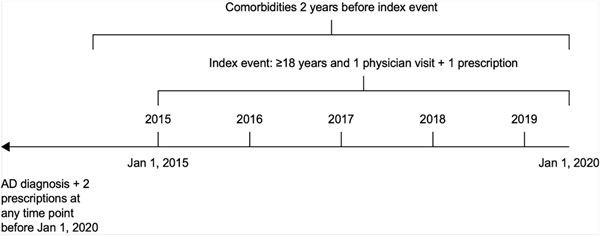

1

**What is already known about this topic?**

- Atopic dermatitis (AD) has high associated disease burden in those affected.
- There are limited data regarding its prevalence and treatment patterns in the adult UK population.

**What does this study add?**

- A better understanding of current prevalence of AD in the UK primary care adult population according to age group and disease severity (mild or moderate‐to‐severe).
- Disease management and secondary care referral patterns in the same patient population.

## 2 INTRODUCTION

Atopic dermatitis (AD) is a common, chronic, inflammatory skin disease, characterised by pruritus and varying, relapsing skin manifestations, leading to disturbed sleep and reduced health‐related quality of life.^1^ AD is often associated with comorbidities, including asthma, conjunctivitis, depression and anxiety.^2^, ^3^ The prevalence of AD in adults varies according to severity, assessment method and geographical region.^1^, ^3^, ^4^, ^5^ In the UK population, estimates for adults affected by the disease range between 1.5% and 9.9%.^6^, ^7^

In the UK, AD is initially managed with emollients or topical corticosteroids to reduce inflammation and flares. Topical calcineurin inhibitors (TCIs) are a second‐line option, initiated by GPs with an extended role or dermatologists/specialists, for flare management and to limit exposure to prolonged steroid use.^7^, ^8^ Intractable/persistent AD was historically managed with systemic immunosuppressant therapies (e.g. methotrexate, ciclosporin, azathioprine and mycophenolate mofetil [MMF]).^9^, ^10^ However, additional treatments are now available as options for these patients, such as the monoclonal antibody dupilumab or Janus kinase inhibitors such as baricitinib.^10^, ^11^, ^12^ In addition, primary care patients may inappropriately be prescribed oral glucocorticoids.^13^ Most UK patients with AD (97%) are treated by GPs, with approximately 11% requiring referral to a specialist.^14^

Despite the high disease burden associated with AD,^1^ data on disease prevalence and treatment patterns in the adult UK population are limited. Electronic healthcare records used by GPs, such as the Clinical Practice Research Datalink (CPRD), provide an opportunity to use real‐world data to investigate the prevalence, comorbidities, affected age groups, current treatment patterns and other factors associated with AD.^14^

The primary objective of this study was to calculate the current prevalence of AD in the UK primary care adult population according to age group and by disease severity (mild or moderate‐to‐severe) from the CPRD database. Patient characteristics and current treatment and secondary care referral patterns for the same patient population by severity of AD (mild vs. moderate‐to‐severe) were also described.

## 3 PATIENTS AND METHODS

### 3.1 Data source

This was a non‐interventional, retrospective, observational study conducted using data from the CPRD database.^15^ The CPRD covers approximately 7% of the UK general population, is broadly representative with respect to demographics, diagnosis, prescriptions and secondary care referrals.^16^ Data for this analysis were extracted from the CPRD GOLD dataset, which includes data from primary care practices using the Vision software system (Cegedim Healthcare Solutions, London, UK).

### 3.2 Study design

**FIGURE 1:**
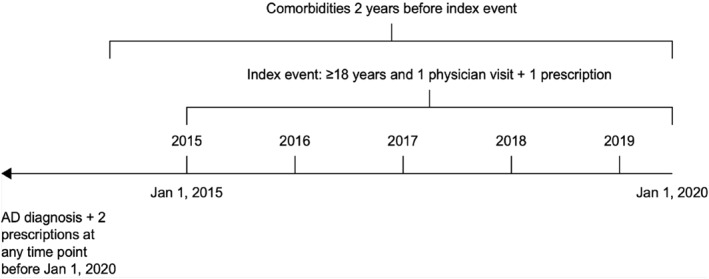
Study time periods. AD, atopic dermatitis.

Study time periods are presented in Figure 1. Patients with AD aged ≥18 years were identified through an established algorithm based on diagnosis code and pharmaceutical treatment (Tables S1 and S2).^14^ Patients with active AD were identified by an AD‐related prescription or AD‐related GP visit within the same calendar year. Moderate‐to‐severe disease was classified as either referral to a specialist (dermatologist or immunopathologist) or a prescription(s) for TCIs, phototherapy, or systemic treatments, including methotrexate/methotrexate sodium, azathioprine, MMF/mycophenolate sodium, ciclosporin and dupilumab. For this analysis, oral glucocorticoids were not considered systemic therapy when identifying patients with moderate‐to‐severe disease, an approach similar to that taken in other studies.^6^, ^7^ Mild AD was classified as disease not meeting these criteria.

Patient demographics and disease‐related factors (type of AD, time since diagnosis and comorbidities) were assessed in the AD UK adult population as of 1 January 2020 for the calendar year 2019. Although data concerning all comorbidities recorded within 2 years before the index event were documented, this analysis included only those considered of special interest^17^ or those commonly reported in other publications (e.g., conjunctivitis and asthma); the index event was defined as a physician visit or prescription during 1 January 2015 to 1 January 2020. Treatment and referral patterns were analysed for patients meeting criteria for active AD in 2019.

### 3.3 Statistical analysis

Baseline characteristics were descriptively analysed and summarised as *N* (%) (categorical variables) or mean ± standard deviation (SD) (continuous variables). Prevalence of AD was calculated as the number of patients meeting criteria for active AD on 1 January of each year (2016–2020) divided by the number of adults in the current CPRD population on that date, expressed as a percentage. Point prevalence on 1 January of each year was estimated by assessing the number of patients in each previous calendar year (e.g. point prevalence as of 1 January 2020 was represented by the prevalence in 2019). Treatment patterns were expressed as the number (%) of patients receiving topical and/or systemic treatments in 2019, and the numbers (%s) with referral of any type (secondary care or specialist treatment) or referral to a specialist likely to treat AD (National Health Service [NHS]/Family Health Service Authority dermatology or NHS immunopathology). No imputation was performed for missing data.

### 3.4 Ethical review and regulatory considerations

This study was conducted in accordance with ethical principles of the Declaration of Helsinki and Good Pharmacoepidemiology Practices, and in accordance with applicable laws and regulations of the UK. Institutional Review Board approval for non‐interventional studies is not required.

## 4 RESULTS

### 4.1 Patient demographics

As of 1 January 2020 (i.e. 2019 prevalence), 72 013 adults in the UK had active AD (Table 1). Most were female (58.2%), mean disease duration was 9.4 ± 5.9 years and mean age was 52.6 ± 21.0 years (Table 2).

**TABLE 1:** Distribution of patients according to atopic dermatitis (AD) severity in the UK adult population, prevalence from 2015 to 2019.

AD severity	2015	2016	2017	2018	2019
	*N* = 121 176	*N* = 100 519	*N* = 86 812	*N* = 79 095	*N* = 72 013
	*n* (%)	*n* (%)	*n* (%)	*n* (%)	*n* (%)
Mild	112 078 (92.5)	92 770 (92.3)	80 135 (92.3)	72 901 (92.2)	66 025 (91.7)
Moderate‐to‐severe	9098 (7.5)	7749 (7.7)	6677 (7.7)	6194 (7.8)	5988 (8.3)

*Note*: *N* represents the total number of patients with AD. *n* represents the number of patients in each AD category for each year.

**TABLE 2:** Demographics and clinical characteristics of patients with atopic dermatitis (AD) as of 1 January 2020. Results are for the calendar year 2019.

Baseline characteristic	Mild AD (*N* = 66 025)	Moderate‐to‐severe AD (*N* = 5988)	Total (*N* = 72 013)
Mean age, years	52.6 ± 21.1	52.3 ± 20.2	52.6 ± 21.0
Female	38 340 (58.1)	3575 (59.7)	41 915 (58.2)
Mean disease duration, years	9.3 ± 5.9	9.5 ± 6.0	9.4 ± 5.9
BMI, kg/m^2^	29.2 ± 6.9	28.4 ± 6.5	29.1 (6.9)
Comorbidities			
Asthma^a^	13 478 (20.4)	1248 (20.8)	14 726 (20.5)
Depression^a^	9012 (13.7)	716 (12.0)	9728 (13.5)
Anxiety^a^	4389 (6.7)	348 (5.8)	4737 (6.6)
Conjunctivitis^a^	2004 (3.0)	186 (3.1)	2190 (3.0)
Country			
England	17 918 (27.1)	1766 (29.5)	19 684 (27.3)
Northern Ireland	6301 (9.5)	604 (10.1)	6905 (9.6)
Scotland	20 684 (31.3)	1761 (29.4)	22 445 (31.2)
Wales	21 122 (32.0)	1857 (31.0)	22 979 (31.9)

*Note*: Results are presented as mean ± SD or *n* (%).

Abbreviations: BMI, body mass index; SD, standard deviation.

^a^ Based on a diagnostic code recorded within 2 years before the date of active disease in 2015–2019.

### 4.2 Prevalence and severity of AD

**FIGURE 2:**
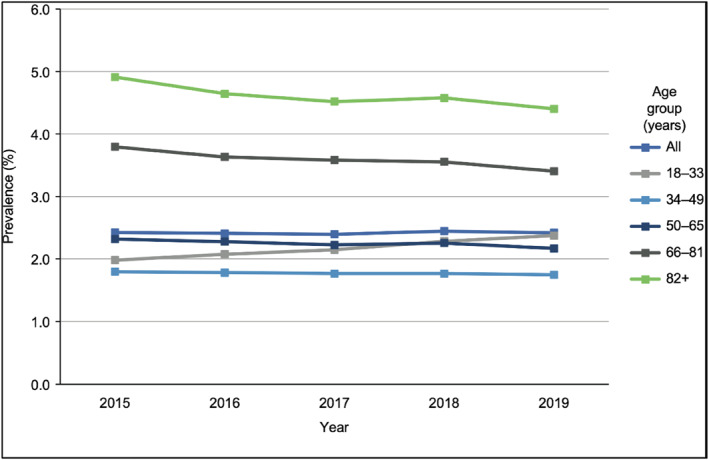
Prevalence of AD in the UK adult population from 2015 to 2019, by age group. AD, atopic dermatitis.

Prevalence of active AD in the overall population remained stable at 2.4% during the period 2015–2019 (Figure 2). Prevalence was highest in those aged ≥82 years (4.4−4.9%) and lowest in those aged 34–49 years (1.7−1.8%). In the cohort of patients with active AD, 7.5%–8.3% had moderate‐to‐severe AD during the 5‐year period (Table 1). There was a linear decrease each year in the CPRD population with AD, and in the number of patients with moderate‐to‐severe AD (from 9098 in 2015 to 5988 in 2019). A similar decrease in patient numbers was also observed in the total CPRD population.

### 4.3 Treatment patterns

The most prescribed treatments in 2019 were topical regimens (in 86.3% of all patients). More AD patients with mild (79.5%) than moderate‐to‐severe (66.9%) disease received topical corticosteroids (Table 3).

**TABLE 3:** Treatment characteristics of patients with atopic dermatitis (AD) as of 1 January 2020. Results are for the calendar year 2019.

Treatment characteristics	Mild AD (*N* = 66 025)	Moderate‐to‐severe AD (*N* = 5988)	Total (*N* = 72 013)
Topical treatment	57 693 (87.4)	4468 (74.6)	62 161 (86.3)
Topical corticosteroids	52 510 (79.5)	4004 (66.9)	56 514 (78.5)
Emollients	4874 (7.4)	221 (3.7)	5095 (7.1)
Topical calcineurin inhibitor	0 (0)	1577 (26.3)	1577 (2.2)
Oral glucocorticoids	13 758 (20.8)	1422 (23.8)	15 180 (21.1)
Systemic treatment (excluding oral glucocorticoids)	–	2200 (36.7)	2200 (3.1)
Methotrexate	–	1218 (20.3)	1218 (1.7)
Azathioprine	–	700 (11.7)	700 (1.0)
Mycophenolate	–	227 (3.8)	227 (0.3)
Ciclosporin	–	93 (1.6)	93 (0.1)
Dupilumab	–	3 (0.05)	3 (0.004)
Oral glucocorticoids and other systemic treatments^a^	_	696 (11.6)	696 (1.0)
Referral of any type at any time in 2019	21 312 (32.3)	3582 (59.8)	24 894 (34.6)
Specialist referral at any time in 2019^b^	–	2508 (41.9)	2508 (3.5)
Number of years from diagnosis to specialist referral^b^	–	8.2 ± 6.2	–

*Note*: Results are presented as mean ± SD or *n* (%).

Abbreviations: –, not applicable due to the algorithm rules; SD, standard deviation.

^a^ Each could be used at any time in 2019, separately or concurrently.

^b^ Specialist referral: National Health Service (NHS) or Family Health Service Authority dermatology, or NHS immunopathology referral.

Around one‐third (36.7%) of patients with moderate‐to‐severe AD were prescribed systemic agents, most frequently methotrexate (20.3%) (Table 3). Oral glucocorticoids were prescribed to 20.8% of patients with mild AD and 23.8% of patients with moderate‐to‐severe AD. The frequency of AD prescriptions during 2019 also indicated that patients were more likely to receive prescriptions for topical treatment than for other treatments in both disease severity groups (Table 4).

**TABLE 4:** Atopic dermatitis (AD) prescription frequency during 2019, by disease severity.

	1–2 prescriptions	3–5 prescriptions	6–8 prescriptions	≥9 prescriptions	No prescriptions
Topical treatment prescriptions per patient					
Mild AD (*N* = 66 025)	39 697 (60.1)	10 952 (16.6)	3707 (5.6)	3337 (5.1)	8332 (12.6)
Moderate‐to‐severe AD (*N* = 5988)	2190 (36.6)	1129 (18.9)	482 (8.1)	667 (11.1)	1520 (25.4)
Oral glucocorticoid prescriptions per patient					
Mild AD (*N* = 66 025)	9904 (15.0)	2080 (3.2)	775 (1.2)	999 (1.5)	52 267 (79.2)
Moderate‐to‐severe AD (*N* = 5988)	777 (13.0)	266 (4.4)	164 (2.7)	215 (3.6)	4566 (76.3)
Systemic treatment prescriptions per patient					
Mild AD (*N* = 66 025)	−	−	−	−	66 025 (100)
Moderate‐to‐severe AD (*N* = 5988)	288 (4.8)	421 (7.0)	492 (8.2)	999 (16.7)	3788 (63.3)

*Note*: Results are presented as *N* (%). Results are recorded anytime during 2019 concomitantly or separate timepoints.

Abbreviation: –, not applicable due to the algorithm rules.

### 4.4 Referral patterns

Almost twice as many patients with moderate‐to‐severe versus mild AD had a referral of any type, which included specialist referrals for AD (59.8% vs. 32.3%, respectively; Table 3). The mean time between diagnosis and dermatology/immunopathology specialist referral for patients with moderate‐to‐severe AD was 8.2 ± 6.2 years. Of 24 894 patients with a referral, 54.3% were aged 50–81 years and 17.0% were aged 18–33 years (Table 5). Disease duration was ≥9 years in approximately 50% of both referred and non‐referred patients. Comorbidities and treatment patterns (other than greater use of oral glucocorticoids in referred [26.8%] vs. non‐referred patients [18.1%]) were similar in the two groups.

**TABLE 5:** Patient, disease and treatment characteristics by referral of any type in 2019.

Characteristics	No referral (*N* = 47 208)	Referral^a^ (*N* = 24 894)
Age group		
18–33 years	13 523 (28.7)	4208 (17.0)
34–49 years	9940 (21.1)	4204 (17.0)
50–65 years	10 827 (22.9)	6146 (24.7)
66–81 years	9601 (20.3)	7360 (29.6)
≥82 years	3317 (7.0)	2887 (11.6)
AD disease duration		
<1 year	4133 (8.8)	2476 (10.0)
1–<3 years	4817 (10.2)	2689 (10.8)
3–<5 years	4643 (9.8)	2455 (9.9)
5–<7 years	4756 (10.1)	2452 (9.9)
7–<9 years	4740 (10.0)	2415 (9.7)
≥9 years	24 119 (51.1)	12 318 (49.7)
Comorbidities		
Asthma	9485 (20.1)	5241 (21.1)
Depression	5735 (12.2)	3993 (16.1)
Anxiety	2688 (5.7)	2049 (8.3)
Conjunctivitis	1290 (2.7)	900 (3.6)
Topical treatment		
Topical corticosteroids	37 528 (79.5)	18 986 (76.5)
Emollients	3436 (7.3)	1659 (6.7)
Topical calcineurin inhibitor	1005 (2.1)	572 (2.3)
Oral glucocorticoids	8540 (18.1)	6640 (26.8)
Systemic and oral glucocorticoid treatment	400 (0.9)	296 (1.2)
Systemic treatment		
Methotrexate	772 (1.6)	446 (1.8)
Azathioprine	478 (1.0)	222 (0.9)
Mycophenolate	147 (0.3)	80 (0.3)
Ciclosporin	64 (0.1)	29 (0.1)
Dupilumab	1 (0.002)	2 (0.008)
AD‐related primary care visits in 2019		
1	6848 (14.5)	3637 (14.7)
2	727 (1.5)	474 (1.9)
3	134 (0.3)	105 (0.4)
4	32 (0.1)	44 (0.2)
≥5	25 (0.1)	19 (0.1)

*Note*: Results are presented as *n* (%).

Abbreviation: AD, atopic dermatitis.

^a^ Number of referrals of any type with a diagnosis over the calendar year 2019: secondary care or specialist treatment.

## 5 DISCUSSION

Our analysis shows that the prevalence of AD in the adult UK population captured in the CPRD remained stable at 2.4% throughout 2015–2019. Both total number of patients with AD (nominator) and total number of patients in the CPRD (denominator) decreased between 2015 and 2019, explaining the lack of impact of decreasing AD patient numbers on prevalence estimates. Based on the criteria used in our study, less than 1 in 10 of the adult AD population had moderate‐to‐severe disease. This is similar to a prevalence of 2.5% (95% confidence interval, 2.2%–2.8%)^5^ in a UK study based on self‐reported AD and to a period prevalence 2008–2018 of 2.0% within the same UK primary care population.^3^ As noted by others,^6^ the true UK population prevalence may be underestimated in the CPRD as mild AD may not be captured by physicians and over‐the counter drugs are not included in medical records. The prevalence reported in our study is lower than the 6.9% reported in another study for the period 1994–2013,^16^ a discrepancy possibly due to use of a different algorithm to define AD, which could impact cohort numbers. In line with other studies,^5^, ^7^, ^18^ we included oral glucocorticoids in the algorithm for the AD population but excluded them from the list of systemic treatments used to define moderate‐to‐severe AD, while including TCIs in the definition. The approach to include or exclude these treatments in the definition of moderate‐to‐severe AD varies across different studies and warrants caution when comparing across cohorts. It should also be noted that the distribution of patients with AD across the UK included in CPRD Gold is uneven, reflecting a higher number of included GP clinics in Scotland and Wales than in England.^16^

Similar to other studies,^6^, ^7^ we observed an increase in AD prevalence with age, which may be attributed to a greater use of healthcare services by the elderly. However, the prevalence of 3.4%–4.9% (see Figure 2) seen in patients aged 66 years and above is lower than that reported in older patients in other studies (e.g. 8.7% in those aged ≥75 years and 4.9%–9.9% in those aged ≥60 years^6^, ^7^). Although prevalence of AD was stable during 2015–2019, the proportion of patients with moderate‐to‐severe AD increased slightly. A possible explanation could be that the introduction of new systemic treatments for moderate‐to‐severe AD during this period resulted in increased identification of patients with severe disease as a consequence of improved education on the disease and diagnosis associated with the greater number of available treatments.

Comorbidities (e.g. asthma, depression, anxiety and conjunctivitis) were less frequent in our study than in others. A descriptive population‐based database analysis conducted using CPRD and Hospital Episode Statistics‐linked data between 2008 and 2018 revealed depression and conjunctivitis in 22.7% and 22.4% of patients, respectively.^3^ Similarly, another retrospective CPRD database analysis of patients with AD (median follow‐up 5.1 years), reported anxiety in 16.8% and depression in 24.6%.^18^ Lower rates of depression (13.5%), anxiety (6.6%) and conjunctivitis (3.0%) in our study may be attributed to data being collected for comorbidities within 2 years prior to a record of active AD.

Topical corticosteroid use in more than three‐quarters of the AD study population is similar to that reported by others, as is low use of TCIs.^7^ Approximately 20% of the AD study population were prescribed oral corticosteroids, which is higher than the percentage observed by others (3%–13%).^7^ In line with treatment guidelines, patients with moderate‐to‐severe AD were prescribed systemic agents (36.7%), most frequently methotrexate (20.3%).

More patients with moderate‐to‐severe AD (59.8%) than mild disease (32.3%) were referred to healthcare services outside primary care in 2019. As these referrals included specialist referrals for AD, which were part of the classification criteria for patients with moderate‐to‐severe AD in the validated algorithm we used, this finding is not unexpected. In our study, treatment patterns were similar between referred and non‐referred patients with AD in 2019, other than greater use of oral glucocorticoids in referred patients. In a study of secondary healthcare data among Danish patients with AD, use of systemic corticosteroids increased in the months preceding the initial hospital visit and declined considerably thereafter.^13^ This interesting trend affirms the patient journey towards systemic treatment, with increased frequency of topical and oral steroid prescriptions among those with moderate‐to‐severe AD. Although the proportion of patients prescribed systemic treatment was lower after than before referral in the current study, this is unlikely to reflect real‐world prescribing as the CPRD does not routinely capture secondary care prescriptions. However, a proportion of secondary care prescribing may be captured from geographical regions in which there is a shared care arrangement whereby systemic non‐biologic drugs are initiated in secondary care and then prescribed by GPs.

We evaluated prescription patterns within the same year of dermatology/immunopathology specialist referral, but did not evaluate temporal patterns between time of referral and time of prescription. The recent availability of new biological treatments for AD is likely to change referral patterns and prescribing behaviour, emphasising the importance of continuous monitoring of prescribing patterns using high‐quality secondary healthcare data.

Strengths of this study include the use of data from one of the UK's largest primary care databases—the CPRD,^15^ which contains medical records for over 11.3 million patients managed by 674 GPs and is representative of the UK general population in terms of demographics. Study limitations include missing data on prescribing activity from specialists from the CPRD, and that the algorithm used for defining moderate‐to‐severe disease might have missed some patients. Patients with moderate‐to‐severe symptoms, but no ready access to specialist care, would be missed in the algorithm as a prerequisite for prescription of systemic treatment is referral to dermatology/immunopathology specialist care. As this was a secondary analysis of existing data (i.e. data were recorded for purposes other than this analysis), there is a risk of selection bias due to data availability and misclassification. Finally, the first biologic, dupilumab, was approved for use in the UK in 2017 and since then an additional biologic, tralokinumab, as well as the oral Janus kinase inhibitors baricitinib, upadacitinib and abrocitinib, have also been approved for patients with moderate‐to‐severe AD. The years covered by this analysis therefore precede widespread uptake of these therapies and may not reflect current prescribing practices for patients with moderate‐to‐severe disease.

## 6 CONCLUSION

The prevalence of active AD in the adult UK population captured in the CPRD was stable during the period 2015–2019 at around 2.4%, and was comparable to estimates from similar studies based on UK primary healthcare records. The mean time between diagnosis and dermatology/immunopathology specialist referral for patients with moderate‐to‐severe AD was 8.2 years. As a chronic condition, AD imposes a substantial impact on the UK healthcare system. The monitoring of prescribing and disease management patterns for AD in the UK primary care setting may help inform future AD treatment guidelines, ultimately leading to better patient outcomes.

## CONFLICT OF INTEREST STATEMENT

CEK has received honoraria, research funding and/or consultancy fees from AbbVie, Almirall, Eli Lilly and Company, Janssen, La‐Roche Posay, Leo, Novartis, Pfizer and UCB. RMcK, AM, BG and L‐BvA are employees of Eli Lilly and Company.

## AUTHOR CONTRIBUTIONS

**C. Elise Kleyn**: Methodology (Supporting); Validation (Equal); Writing – review & editing (Equal). **Robert McKenzie**: Methodology (Supporting); Validation (Equal); Writing – review & editing (Equal). **Alexandra Meeks**: Formal analysis (Lead); Methodology (Equal); Writing – review & editing (Supporting). **Beatrice Gittens**: Conceptualization (Supporting); Formal analysis (Supporting); Validation (Equal); Writing – review & editing (Equal). **Lill‐Brith von Arx**: Conceptualization (Lead); Formal analysis (Equal); Funding acquisition (Lead); Methodology (Equal); Resources (Lead); Supervision (Lead); Visualization (Equal); Writing – review & editing (Equal).

## ETHICS STATEMENT

Institutional Review Board approval for non‐interventional studies is not required.

## Supporting information

Supporting Information S1

## Data Availability

The study uses data from the Clinical Practice Research Datalink (CPRD). CPRD does not allow the sharing of patient‐level data.
