# Supplementary material for: Prevalence and treatment patterns of adult atopic dermatitis in the UK Clinical Practice Research Datalink
Source: Skin Health Dis. 2023 May 10;3(4):e232. doi: 10.1002/ski2.232 (PMC10395620; doi:10.1002/ski2.232)
Supplement: Supplementary file 1 — Supporting Information S1 [file SKI2-3-e232-s001.docx]

**Supporting Information**

**Table S1** Data collection schedule through an established algorithm based on diagnosis code

**1. Atopic dermatitis (AD) coding algorithm:**

An established algorithm based on diagnosis code and pharmaceutical treatment identified adult (age ≥18) patients with AD (Abuabara *et al*. 2017^a^; Lowe *et al*. 2020^b^).

| AD/eczema | M111.00 |
| --- | --- |
| Infantile eczema | M112.00 |
| Flexural eczema | M113.00 |
| Allergic/intrinsic eczema | M114.00 |
| Eczema not otherwise specified | M12z100 |

**PLUS at least two treatment codes for any skin-directed therapy on separate dates** (at any timepoint relative to the AD diagnosis, because symptoms may precede the actual diagnosis).

**2. At least two prescription codes for skin treatments (see Table S2)**: 1) Phototherapy; and 2) primary care prescriptions for *topical* emollients, corticosteroids, or calcineurin inhibitors, or *oral* corticosteroids, azathioprine, methotrexate, cyclosporine, or mycophenolate (the prescription list used by Lowe *et al*. 2020^b^ differs from Abuabara *et al*. 2017^a^).

^a^Abuabara K, Magyari AM, Hoffstad O, *et al*. Development and validation of an algorithm to accurately identify atopic eczema patients in primary care electronic health records from the UK. *J Invest Dermatol* 2017; 137: 1655–62.

^b^Lowe KE, Mansfield KE, Delmestri A, *et al*. Atopic eczema and fracture risk in adults: a population-based cohort study. *J Allergy Clin Immunol* 2020; 145 (2): 563–71.E8.

**Table S2** Atopic dermatitis treatment codes

| British National Formulary Code | Description |
| --- | --- |
| 13.00.00.00 | Skin |
| 13.01.00.00 | Management of skin conditions |
| 13.01.01.00 | Vehicles |
| 13.02.00.00 | Emollient and barrier preparations |
| 13.02.01.00 | Emollient skin preparations |
| 13.02.01.01 | Emollient bath additives and shower preparations |
| 13.02.02.00 | Barrier preparations |
| 13.03.00.00 | Topical local anaesthetic and antipruritic |
| 13.04.00.00 | Topical corticosteroids |
| 13.05.00.00 | Preparations for eczema and psoriasis |
| 13.05.01.00 | Preparations for eczema |
| 13.05.03.00 | Drugs affecting the immune response |
| 08.01.03.00 | Antimetabolites (Methotrexate) |
| 08.02.01.00 | Antiproliferative immune suppressants (Azathioprine, Mycophenolate) |
| 08.02.02.00 | Other immunosuppressants (Cyclosporin, Tacrolimus) |
| 13.10.01.01 | Antibacterial preparations for skin |
| 13.10.01.02 | Antibacterial preparations for skin |
| 13.10.03.00 | Antiviral preparations for skin |
| 13.05.03.00 | Drugs affecting the immune response |
| 14.05.00.00 | Interferon gamma |
| READ Phototherapy Code | Description |
| 863..11 | Phototherapy |
| 8631.00 | Phototherapy |
| 8632.11 | PUVA/phototherapy |
| 8632.14 | UVB phototherapy |
| 8632.15 | Ultraviolet light phototherapy |
| 7G0E.00 | Phototherapy to skin |
| 7G0E200 | Combined photochemotherapy and UVA light therapy to skin |
| 7G0E300 | Combined photochemotherapy and UVB light therapy to skin |
| 7G0Ey00 | Other specified phototherapy to skin |
| 7G0Ez00 | Phototherapy to skin NOS |
| 863..00 | Phototherapy/radiation therapy |
| 863Z.00 | Radiation/phototherapy NOS |
| Z6E1.12 | Ultraviolet light phototherapy |
| Z6E1111 | UVA phototherapy |
| Z6E1211 | UVB phototherapy |
| Z6E1411 | PUVA phototherapy |
| Z6E2.00 | Photochemotherapy |
| Z6E5.00 | Intermittent phototherapy |

NOS, not otherwise specified; PUVA, Psoralen plus ultraviolet A; UVA, ultraviolet A; UVB, ultraviolet B.
